# Identification of *Mycobacterium avium subsp. paratuberculosis* (MAP) in Sheep Milk, a Zoonotic Problem

**DOI:** 10.3390/microorganisms8091264

**Published:** 2020-08-20

**Authors:** Sepideh Hosseiniporgham, Tiziana Cubeddu, Stefano Rocca, Leonardo A. Sechi

**Affiliations:** 1 Dipartimento di Scienze Biomediche, Università di Sassari, Viale San Pietro 43 b, 07100 Sassari, Italy; hosseini.shp83@gmail.com; 2 Dipartimento di Medicina Veterinaria, Università di Sassari, 07100 Sassari, Italy; tcubeddu@uniss.it (T.C.); rocca@uniss.it (S.R.); 3 Mediterranean Centre for Disease Control, University of Sassari, 07100 Sassari, Italy

**Keywords:** *Mycobacterium paratuberculosis*, JD, ELISA, qPCR, IS900, milk, MqPCR, BTM, SELISA, MELISA, FPCR

## Abstract

Johne’s disease (JD) is a life-threatening gastrointestinal disease affecting ruminants, which causes crucial economical losses globally. This ailment is caused by *Mycobacterium avium subsp. paratuberculosis* (MAP), a fastidious intracellular pathogen that belongs to the Mycobacteriaceae family. This acid-fast, hard-to-detect bacterium can resist milk pasteurization and be conveyed to dairy product consumers. Many studies have emphasized the zoonotic nature of MAP, suggesting an association between MAP and some gastroenteric conditions such as Crohn’s disease in humans. This underlines the importance of utilizing efficient pasteurization alongside a state-of-the-art diagnostic system in order to minimize the possible ways this pathogen can be conveyed to humans. Until now, no confirmatory MAP screening technique has been developed that can reveal the stages of JD in infected animals. This is partially due to the lack of an efficient gold-standard reference method that can properly evaluate the performance of diagnostic assays. Therefore, the following research aimed to compare the merits of qPCR and ELISA assessments of milk for the detection of MAP in a total of 201 Sardinian unpasteurized sheep milk samples including 73 bulk tank milk (BTM) and 128 individual samples from a MAP-infected flock (MIF) applying various reference models. Accordingly, milk qPCR and ELISA assessments, together and individually, were used as reference models in the herd-level study, while serum ELISA and fecal PCR were similarly (together and in isolation) considered as the gold standards in the individual-level diagnosis. This study showed that the type of gold-standard test affects the sensitivity and specificity of milk qPCR and ELISA significantly. At the individual level in the MAP-infected flock, serum ELISA in isolation and together with fecal PCR were recognized as the best references; however, the best correlation was seen between milk and serum ELISA (*p* < 0.0001). Regarding the detection of MAP in BTM, qPCR IS900 was recognized as the most sensitive and specific diagnostic test (*p* < 0.0001) for monitoring the MAP shedders and animals with clinically developed symptoms within herds, under the condition that both milk qPCR and milk ELISA tests formed a binary reference model. The BTM analyses (qPCR and ELISA) revealed that MAP positivity has a seasonal pattern. This hypothesis was proven through a longitudinal study on 14 sheep herds.

## 1. Introduction

Johne’s disease (JD) is a global chronic gastroenteric condition that affects ruminants, causing the dairy industry serious economic concern [1,2]. JD is triggered by *Mycobacterium avium subsp. paratuberculosis* (MAP), an acid-fast bacterium that belongs to the Mycobacteriaceae family [2]. Many studies have emphasized the association of MAP with other gastrointestinal conditions such as Crohn’s disease (CD) in humans due to the symptomatic similarities that CD has in common with JD [3,4,5,6]. Animals that are infected with this bacterium are predisposed to other infections and conditions such as lameness, mastitis, and pneumonia due to JD causing an incurable immunodeficiency [2]. Johne’s disease has three main stages—silent, subclinical, and clinical. The symptom manifestation usually begins after a long latency period (around 2 to 10 years). Therefore, the infected animals remain as reservoirs for the rest of their lives or transmit the disease to healthy individuals, either by direct contact and shedding of the MAP into the environment (horizontal transmission) or by reproducing newborn lambs (vertical transmission) [2,7].

Different MAP strains may infect different ruminants [1]. Sheep are susceptible to both S and C MAP types, whereas cattle are resistant to the S type [8]. These types differ not only by genotypic characteristics, but also by phenotypic divergence in pigmentation, iron metabolism, cytokine induction, and transcriptional patterns, as shown in experiments performed on a human monocyte cell line model (THP-1) [1]. MAP strains belonging to the S type are notorious for their fastidious and slow-growing profiles, making the detection of this bacterium by some routine laboratory methods more laborious [1,9].

In Sardinia, unpasteurized sheep’s milk is usually used for cheesemaking purposes, which raises concerns over the consumption of these products and increases the possibility of some MAP-relevant diseases occurring in people in this region. This underlines the importance of using an efficient and confirmatory monitoring assay that can recognize the infected animals prior to using their milk in the cheese production cycle.

MAP has been reported at the herd level by testing various sample types, such as tissue, feces, and milk [10,11]. As milk can feasibly be manipulated, while blood and fecal samples cannot, milk is considered a potent specimen for diagnosing diseases relevant to ruminants [12,13].

Interestingly, the bulk tank milk (BTM) test is one the most economical and efficient herd-level analyses for screening of JD, which provides a general overview of the health status of the animals in each herd, especially as the presence of asymptomatic animals can lead to underestimation of the sensitivity and specificity of the diagnostic assays [14,15]. Evidence of MAP was discovered in milk and feces cultured from both symptomatic and asymptomatic carriers [16,17].

Culturing is one of the most reliable and specific techniques commonly used as a gold-standard reference in MAP diagnostic studies of milk and fecal samples. However, it lacks sensitivity (30–50%), partly due to the prolonged incubation time of between 7 and 16 weeks on solid media [9,10,18,19]. In addition, the concentrations of MAP (especially viable ones) in milk and colostrum are naturally lower than in other samples, such as feces [9]. Although pretreatment of milk samples could augment the load of the bacterium in such samples, some preparation steps, such as chemical decontamination and incubation with antimicrobial brews, sacrifice the sensitivity of culture-based assessments for more specificity [9]. This highlights the importance of applying alternative gold-standard approaches that increase the sensitivity and specificity of the diagnostic assay simultaneously.

The advent of the quantitative polymerase chain reaction (qPCR) technique was a great revolution in the area of molecular diagnosis that led to remarkable improvements in the detection and quantification of MAP DNA in milk and other clinical samples. Among the advantages of the qPCR technique are its availability, sensitivity, reproducibility, rapidity, and cost-effectiveness. Until now, some MAP sequences such as F57, IS-MAP02, and hspX have been targeted for qPCR analysis; however, the insertion sequence IS900, due to its high sensitivity (12–18 copies in the total MAP genome) and specificity, has been adopted in MAP screening programs [9,20,21,22].

On the other hand, the enzyme-linked immunosorbent assay (ELISA) is commonly used to assess the presence of sera antibodies directed against MAP in infected animal serum [10]. Although some studies have demonstrated that MAP-infected animals develop titers of antibodies against MAP in milk as well as sera [23,24], some studies believe that milk and serum ELISA act imprecisely before the clinical stage of JD [11,14].

However, the sensitivity and specificity of the diagnostic assay (i.e., qPCR, ELISA) could be affected by the type of gold standard assigned for the detection. In addition, the efficiency of a reference test is also influenced by some factors, such as age of the animals, stage of the ailment, and source of the specimen [25]. In contrast to some worldwide studies that used only a single test, such as fecal culture, as the gold standard [25], the following study aimed to compare the efficiency of various gold-standard reference models of serum ELISA (SELISA) and fecal PCR (FPCR) at the individual level, and milk qPCR (MqPCR) and milk ELISA (MELISA) at the herd level (in isolation and in combination), in order to evaluate the sensitivity and specificity of each milk assay (MqPCR and MELISA) in predicting real MAP-infected cases and prove whether or not milk is a top-of-the-line sample over other specimens to detect MAP in sheep flocks. Moreover, in this study, a high-throughput IS900-based qPCR assay was developed using a modified DNA extraction protocol from low-quantity milk samples (5 mL), and its efficiency was compared with milk ELISA in a longitudinal and low-scale seasonal study as well.

## 2. Materials and Methods

### 2.1. Bacterial Strains

*Mycobacterium avium subsp. paratuberculosis* strain 1515 (ATCC 43015) and *Mycobacterium smegmatis* strain MC^2^155 (ATCC 700084) were used as positive and negative controls through the study (RIVM, Bilthoven, The Netherlands). They were grown in Middlebrooke 7H9 broth (Sigma-Aldrich, Milan, Italy) supplemented with 10% Oleic Albumin Dextrose Catalase (OADC; Sigma-Aldrich, Milan, Italy) and 2 mg mycobactin J (Allied Monitor, Fayette, MO, USA) and incubated at 37 °C for 3 days to 4 weeks (depending on the mycobacterial species).

### 2.2. Sample Collection

Raw sheep milk is commonly used in the cheese production process in Sardinia. A total of 201 unpasteurized sheep milk samples, including 73 bulk tank milk (BTM) samples from 59 dairy herds and 128 individual milk samples, were used in this study. The 128 individual samples were certified by the Veterinary Department of the University of Sassari and collected from a MAP-infected flock (MIF) of Northern Sardinia (Italy) (Table 1). BTM sampling started from July 2018 and continued in different intervals until July 2019. Of the 59 herds, 14 herds were selected for a longitudinal study and monitored twice by BTM analyses (qPCR and ELISA) on different dates. The samples were taken in a sterile condition, kept at 4 °C during transportation, divided into aliquots immediately after transferring to the diagnostic laboratory, and stored at −80 °C without using any preservatives.

### 2.3. DNA Extraction from BTMs and MIF Milk Samples (MIFMs)

A modified DNA extraction protocol was established based on minimizing the quantity of milk samples (5 mL) required for DNA extraction and adding some milk preparation steps prior to DNA extraction as follows: dilution with phosphate-buffered saline (PBS; pH = 7.4; up to 30 mL), treatment with 0.75% (*w/v*) hexadecylpyridinium chloride (HPC; Sigma-Aldrich, Milano, Italy), and homogenization by ribolyzer using glass beads (3 mm). Briefly, milk samples were allowed to stand at room temperature (RT) for one hour. Five milliliters of each milk sample was diluted in 25 mL 1× PBS (pH = 7.4) and centrifuged at 5000× *g* rpm and 4 °C for 30 min. Then, the whey phase was decanted, the cream and pellet layers were collected, and DNA was extracted from both layers. At the next step, the cream and pellet were resuspended in 6 mL of hexadecylpyridinium chloride (HPC: Sigma-Aldrich, Milan, Italy; 0.75% *w/v*), vigorously agitated by vortex, and incubated at RT for 1 h. The aim of this step was to release any MAP cells that might be surrounded by a lipid or cream layer in the milk samples. Then, samples were centrifuged at 5000× *g* rpm at 15 °C for 20 min and supernatant including cream residuals and HPC was discarded. Subsequently, pellets were resuspended in 1 mL 1× PBS (pH = 7.4), transferred into specific microtubes, and homogenized by ribolyzer using glass beads (Sigma-Aldrich, Milan, Italy; diameter of 3 mm) for 4 cycles of 45 s at 4 m/s. This step aimed to homogenize the samples and release any MAP cells that might be trapped in somatic cells [26,27]. Then, this suspension was centrifuged at 10,000× *g* rpm and 4 °C for 15 min, and DNA was extracted from the pellet using an RTP Mycobacteria kit (Stratec kit, Stratec Molecular GmbH, Berlin, Germany). The RTP Mycobacteria protocol was modified as follows: NAC buffer (NAC buffer comes with the other RTP-kit reagents and it probably contains N-acetylcysteine that is used for reducing the sputum viscosity) was replaced with 1× PBS (pH = 7.4); incubation times at 95 °C (15 min) and 65 °C (10 min) were increased to 30 and 20 min, respectively; and the column centrifugation steps were extended by 2 min based on the density of samples.

### 2.4. Milk qPCR (MqPCR) 

qPCR has been performed to quantify the presence of IS900 in milk samples. Two mycobacterial species of *Mycobacterium avium subsp. paratuberculosis* strain 1515 and *Mycobacterium smegmatis* strain MC^2^155 were used as the positive and negative controls, respectively, through the qPCR analysis. Autoclaved Milli-Q water and SYBR Select Master Mix (Thermofisher Scientific, Applied Biosystems, Milan, Italy) were also included as the second and third negative controls, respectively. The MAP-specific primers AV1 (5′-ATGTGGTTGCTGTGTTGGATGG-3′) and AV2 (5′-CCGCCGCAATCAACTCCAG-3′) (Sigma-Aldrich, Milan, Italy) were used for amplification of IS900. Two to five microliters of extracted DNA (20–100 ng) were added into each reaction consisting of 10 μL SYBR Select Master Mix, 0.4 μL (10 μM) of each of the primers AV1 and AV2, and water added up to the final volume of 20 μL. The amplification conditions were as follows: initial denaturation at 95 °C for 3 min, followed by 50 cycles of denaturation at 95 °C for 40 s, annealing at 68 °C for 40 s, and extension at 72 °C for 40 s, with a final extension at 72 °C for 5 min.

### 2.5. Milk ELISA (MELISA)

In order to assess the presence of antibodies against MAP in milk samples, an indirect commercial milk ELISA kit (IDEXX Laboratories, Westbrook, ME, USA) was purchased.

As a sample preparation step prior to ELISA, inhibitors such as lipids (cream) and somatic cells (pellet) were removed from milk samples. To do this, samples were centrifuged at 10,000× *g* and 4 °C for 2 min, and then the whey phase, the liquid between the cream and pellet, was aspirated into a new Eppendorf tube (1.5 mL) and stored at −28 °C.

MELISA was performed according to the manufacturer’s (IDEXX) instructions. Briefly, samples (BTMs-MIFMs) were gently inverted few times and diluted (1:2) in dilution buffer N.12, *Mycobacterium phlei* extract (using an uncoated 96-well microplate), in order to reduce cross-reactivity between nonspecific mycobacterial antibodies (abs; other than MAP-abs) and MAP epitopes (coating peptide). The positive and negative controls were also included in the assessment and diluted (1:20) in the same buffer (N.12) as milk samples. Then, they were homogenized using an orbital shaker (100 rpm/5 min) and incubated at RT for 2 h. Afterwards, samples (100 μL) were transferred into the wells of epitope-coated plates and incubated at RT for around 1 h. This step was followed by a washing step (300 μL × 5 times), adding conjugate (1:100, diluted in dilution buffer N.1), incubation at RT for 30 min, a further washing step (300 μL × 3 times), and adding 100 μL substrate 3,3′,5,5′-Tetramethylbenzidine (TMB) to each well. Finally, the optical densities (ODs) were read at the wavelength of 450 nm, converted to sample-to-positive ratios (S/P%) and the results were interpreted accordingly. Samples with S/P% of below 20, between 20–30, and 30 and above were classified as negative, suspect, and positive, respectively.

S/P% = 100 × ((sample OD (450 nm) − negative control OD (450 nm) − (positive control OD (450 nm) − negative control OD (450 nm))

## 3. Statistical Analysis

Statistical analysis was carried out and then authenticated using R and Graphpad Prism 8, respectively. A number of samples tested by MELISA or SELISA produced suspect results. This transformed the dataset features from binary to ternary and might affect the ease of analysis. In order to overcome this data discrepancy, the tests related to these samples were repeated. MELISA-suspect BTMs and MIFMs were subjected to another trial. Regarding the SELISA-suspect MIFMs, samples that had at least two negative test results among MqPCR, MELISA, and FPCR were considered negative.

In order to perform a receiver operating characteristic (ROC) curve test, three different binary-based reference models (0,1) were suggested for both the MAP-infected flock (MIF) and BTM groups. In the MIF group, a binary dataset (0,1) was created based on the results of both SELISA and FPCR (individuals with at least one positive test were considered positive) and adjusted as the first gold-standard model reflecting the positive/negative status of each animal [12,28]. SELISA and FPCR datasets were separately considered as the second and third reference models to present the MAP status of each animal. In the BTM group, since there was no information on the clinical status of each herd animal, three different reference models were suggested based on the results of MqPCR and MELISA. In the first approach, the results of MqPCR and MELISA were interpreted as a binary-format dataset (0,1) and adjusted as a gold standard demonstrating the clinical status of each herd (herds with only one milk assay (MqPCR or MELISA) considered positive) [12]. In the second and third approaches, each of the MqPCR and MELISA datasets were separately converted into binary numbers (0,1) and used as a gold standard to test the efficiency of the other milk assay (MELISA or MqPCR). Then, ROC curve and area under the curve (AUC) analyses were carried out as described before, and the sensitivity and specificity of the reference model was assessed with different thresholds (cutoffs were defined according to the results of MqPCR or MELISA that were continuous variables). In another analysis, the level of dependency between different test results was measured by Chi-square analysis using Graphpad Prism 8. For this purpose in the MIF group, continuous variables (MqPCR-MELISA) were converted to categorical variables (binary: (0,1)) and together with SELISA and FPCR datasets (that were originally categorical variables) used for Chi-square analysis. Regarding BTMs, a Chi-square test was executed on the categorical format of the MqPCR and MELISA datasets.

## 4. Results

### 4.1. BTM qPCR

**Figure 1:**
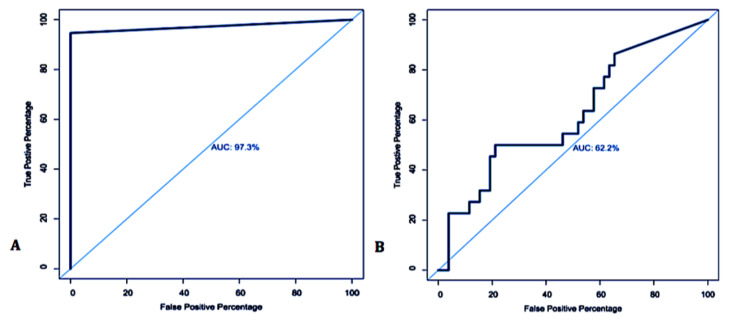
Receiver operating characteristic (ROC) curve analysis and corresponding area under the curve (AUC) analysis of the MqPCR dataset when the reference models were adjusted based on MqPCR and MELISA (**A**) and MELISA (**B**), respectively.

**Figure 2:**
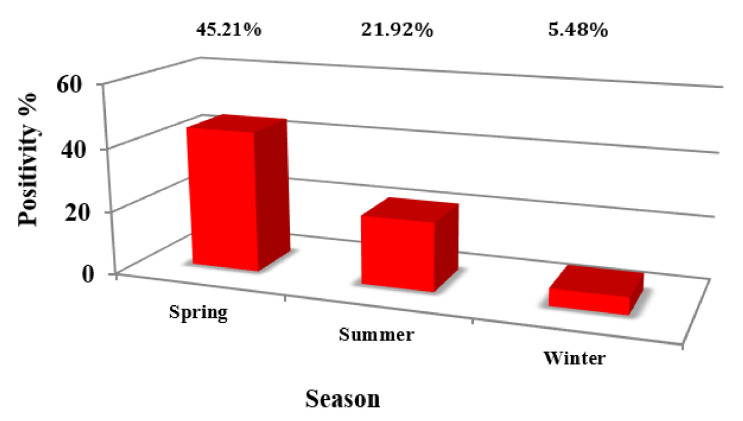
Distribution of MqPCR-positive BTMs in three different seasons: spring (45.21%), summer (21.92%), and winter (5.48%).

**Figure 3:**
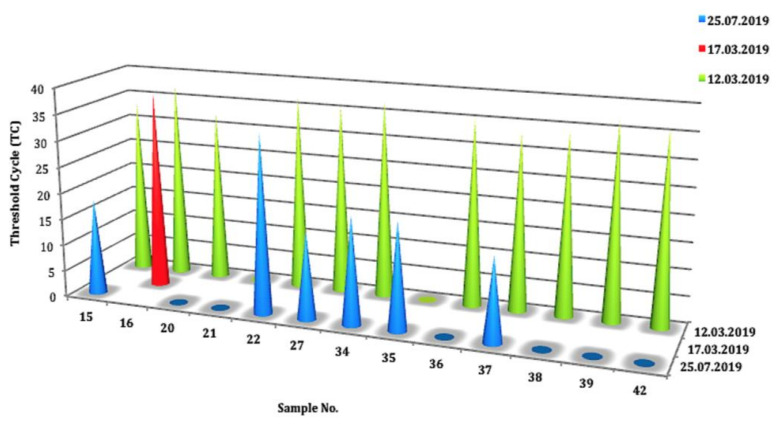
Threshold cycle (TC) changes of BTMs over MqPCR analysis on 14 sheep herds in a longitudinal study (two instances of sampling). At first, 42.86% of BTMs were recognized as positive by qPCR, then changed to negative at the second trial. In total, 28.57% were qPCR-positive in both trials. However, the higher concentrations of DNA (1.05 × 10^2^ ng/μL > C < 5.25 × 10^−6^ ng/μL) corresponding to lower TC values (15–34 cycles) were visible in the second trial.

Of the 73 BTMs, 71.62% showed the evidence of MAP DNA at different threshold cycles (TC), in contrast to 28.38% that were negative. The positivity ratio ranged between 13 to 38 cycles, corresponding to the DNA concentration of 1.05 × 10^2^ ng/μL > C < 5.25 × 10^−6^ ng/μL. ROC curve analysis on the MqPCR dataset showed that the sensitivity and specificity of MqPCR were remarkable when both MqPCR and MELISA were assigned as the gold standard (Approach one: AUC = 0.97, Control vs MAP-infected, cutoff = 6.58, sensitivity: 0.95, specificity: 1, *p* < 0.0001; Figure 1A) compared to when MELISA was adjusted as a reference test (Approach three: AUC = 0.622, control vs MAP-infected, cutoff = 35.42, sensitivity: 0.5, specificity: 0.79, *p* = 0.098; Figure 1B). The results showed that qPCR positivity follows a sensible seasonal pattern, so that spring, with 45.21% positive cases, ranked first, and summer and winter placed after, with 21.92% and 5.48% positive cases, respectively (Figure 2). Furthermore, the longitudinal study on 14 herds with over two instances of sampling (BTM) showed that 21.43% of samples were MqPCR-negative at first, but then became positive; 28.57% and 7.14% were MqPCR-positive and -negative, respectively, across both sampling instances. Conversely, 42.86% showed evidence of MAP positivity at first, but were negative at the second sampling (Figure 3, Table 2). This assessment suggests that some animals among the herds were likely MAP shedders.

### 4.2. BTM ELISA

**Figure 4:**
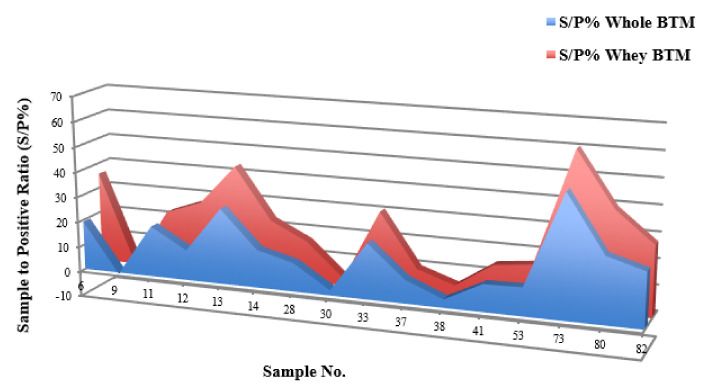
MELISA sample-to-positive ratio (S/P%) of 16 BTMs before and after fractionating milk samples.

In a comparative trial, the impact of the milk preparation step prior to MELISA was evaluated on 15 BTMs. The results demonstrated that by removing cream and pellet fractions from BTMs, sample-to-positive ratios (S/P%) were raised significantly by 0.3 to 15 degrees (Mean~7) (Figure 4, Table 3). This is due to the presence of inhibitors in milk samples concentrated in the cream and pellet layers.

**Figure 5:**
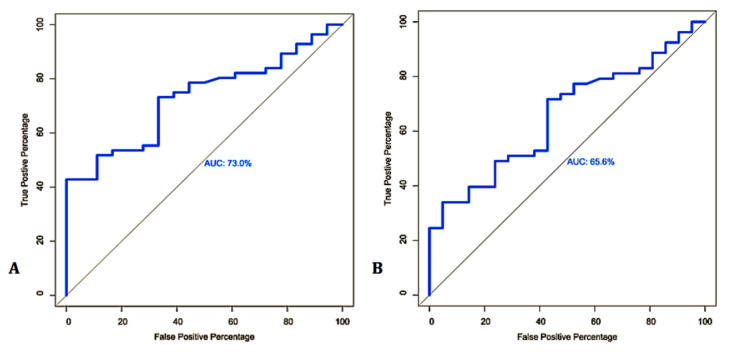
Receiver operating characteristic (ROC) curve analysis and corresponding area under the curve (AUC) analysis of the MELISA dataset when MqPCR and MELISA (**A**) and MqPCR (**B**) were considered as the reference models.

**Figure 6:**
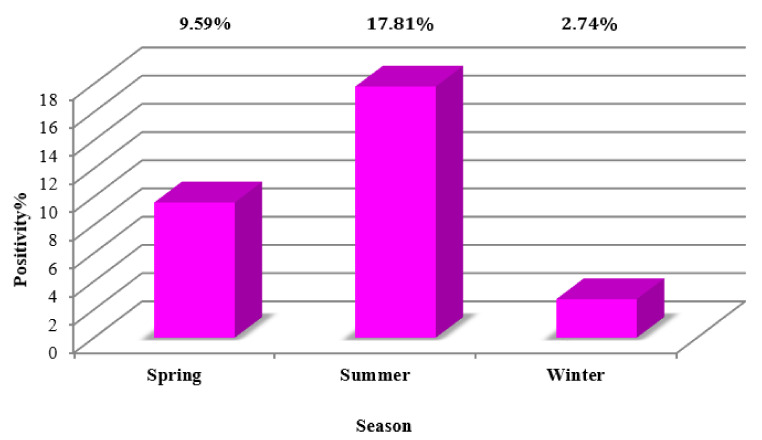
Distribution of MELISA-positive BTMs in three different seasons: spring (9.59%), summer (17.81%), and winter (2.74%).

**Figure 7:**
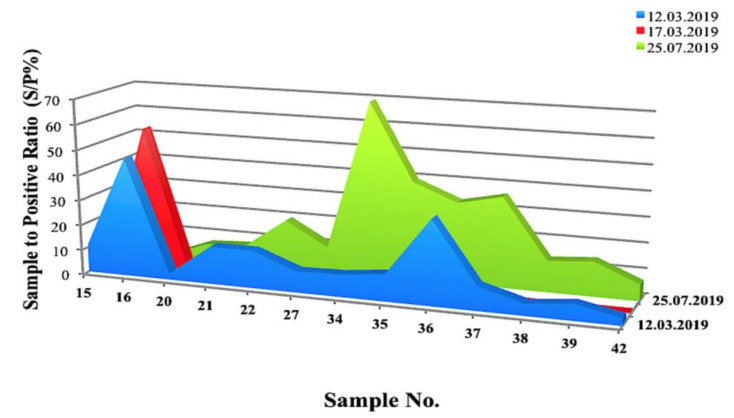
Changes in the MELISA sample-to-positive ratio (S/P%) in a longitudinal study (28 BTMs from 14 sheep herds).

Of the 73 BTMs, 29.7% showed the presence of antibodies against MAP, whereas 70.26% were MELISA-negative. Interestingly, the results of ROC curve analysis showed that MELISA represents the best cutoff, along with an optimal sensitivity and specificity when both MqPCR and MELISA were selected as the gold standard (Approach one: AUC = 0.73, control vs MAP-infected, cutoff = 13.94, sensitivity: 0.59, specificity: 0.89, *p* = 0.00035; Figure 5A) compared to when MqPCR was adjusted as the reference test (Approach two: AUC = 0.656, control vs MAP-infected, cutoff = 33.03, sensitivity: 0.34, specificity: 0.95, *p* = 0.037; Figure 5B). The seasonal pattern was also seen in the ELISA results. However, in contrast to the MqPCR results, the highest number of positive cases was recorded in the summer, with 17.81%, followed by 9.59% and 2.74% in the spring and winter, respectively (Figure 6). The longitudinal study on 14 herds over two instances of sampling demonstrated that 14.28% and 57.14% were MELISA-positive and -negative, respectively, in both evaluations, and that 28.57% were negative at first, but became MELISA-positive later (Figure 7, Table 4). This evaluation was in accordance with the result of the seasonal study and confirmed that antibody titers against MAP have an upward trend in the summer, indicating changes in S/P% ranging from 1.3% to 60.4%.

**Figure 8:**
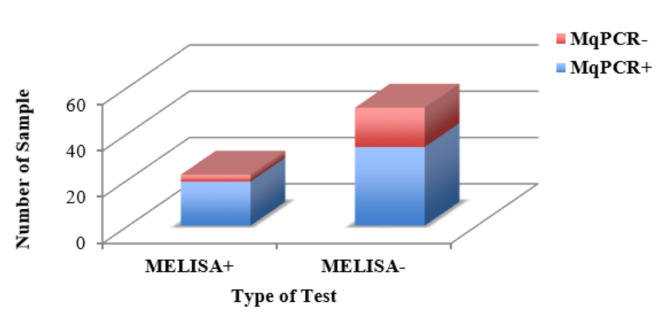
The distribution of 73 BTM samples classified into four binary groups based on the positive/negative status in the MqPCR and MELISA tests. Pos (MqPCR) and Neg (MELISA) constituted the main proportion of samples (46.58%), followed by 26.03% Pos (MqPCR and MELISA), 23.3% Neg (MqPCR and MELISA), and 4.11% Neg (MqPCR) and Pos (MELISA). Pos: positive; Neg: negative.

The Chi-square test showed a significant correlation between MqPCR and MELISA: X^2^ (1, *n* = 73) = 2.99, *p* < 0.1 (26.03% positive (Pos) (MqPCR and MELISA), 46.6% Pos (MqPCR) and negative (Neg) (MELISA), 23.3% Neg (MqPCR and MELISA) and 4.11% Neg (MqPCR) and Pos (MELISA) (Figure 8, Table 5).

### 4.3. MIF MqPCR and MELISA

**Figure 9:**
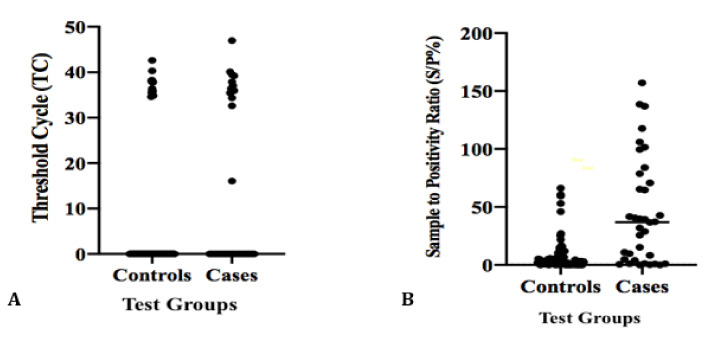
Scatterplots of the distribution of 128 milk samples (MIF) in two categories of healthy controls (HCs) and MAP-infected cases tested by MqPCR (**A**) and MELISA (**B**). HCs were adjusted based on the results of both SELISA and FPCR tests. The threshold cycle corresponding to MqPCR positivity was determined as being between 16 and 46 cycles (**A**), whereas the S/P% corresponding to MELISA positivity was between 31.93% and 157.11% (**B**).

**Figure 10:**
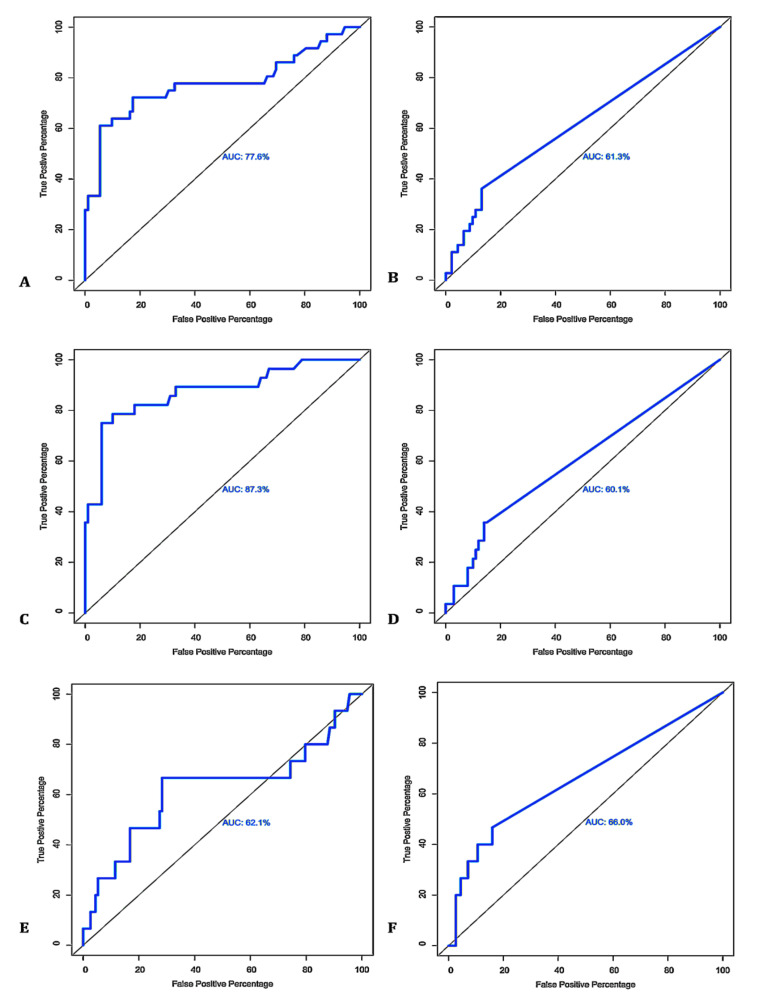
Receiver operating characteristic (ROC) curve analysis and corresponding area under the curve (AUC) analysis of MELISA (**A**,**C**,**E**) and MqPCR (**B**,**D**,**F**) datasets. The sensitivity and specificity of the MELISA and MqPCR tests in the detection of MAP in 128 MIF milk samples were evaluated based on three different gold standards of SELISA and FPCR (**A**,**B**), SELISA (**C**,**D**), and FPCR (**E**,**F**).

Of the 128 MAP-infected flock milk samples, 19.53% and 80.47% were detected as being MqPCR-positive and -negative, respectively. The positivity ratio ranged between 16 and 46 cycles (TC), corresponding to a DNA concentration of 1.05 × 10^2^ ng/μL > C < 5.25 × 10^−6^ ng/μL (Figure 9A). MELISA also produced positivity (21.09%) and negativity (78.91%) rates close to those of MqPCR, whereas the MELISA S/P% ranged from 31.93% to 157.11% (Figure 9B). ROC curve analysis was carried out by comparing the sensitivity (SN) and specificity (SP) of MqPCR and MELISA in various cutoffs based on three different binary reference models. When both SELISA and FPCR were used as the reference model, MELISA was more sensitive and specific (MELISA; AUC = 0.77, control vs MAP-infected, cutoff = 26.72, sensitivity: 0.61, specificity: 0.95, *p* < 0.0001; Figure 10A) than MqPCR (MqPCR; AUC = 0.61, control vs MAP-infected, cutoff = 8.03, sensitivity: 0.4, specificity: 0.87, *p* = 0.047; Figure 10B). MELISA also showed the highest sensitivity and specificity when the binary reference model was based on SELISA only (MELISA; AUC = 0.87, control vs MAP-infected, cutoff = 26.72, sensitivity: 0.75, specificity: 0.94, *p* < 0.0001; Figure 10C) compared to MqPCR (MqPCR; AUC = 0.6, control vs MAP-infected, cutoff = 24.325, sensitivity: 0.36, specificity: 0.86, *p* = 0.1; Figure 10D). However, when the reference model was based on FPCR, the specificity of MELISA slightly dropped by 0.12 (MELISA; AUC = 0.62, control vs MAP-infected, cutoff = 8.20, sensitivity: 0.67, specificity: 0.72, *p* = 0.129; Figure 10E) compared to MqPCR (MqPCR; AUC = 0.66, control vs MAP-infected, cutoff = 8.03, sensitivity: 0.47, specificity: 0.84, *p* = 0.044; Figure 10F).

**Figure 11:**
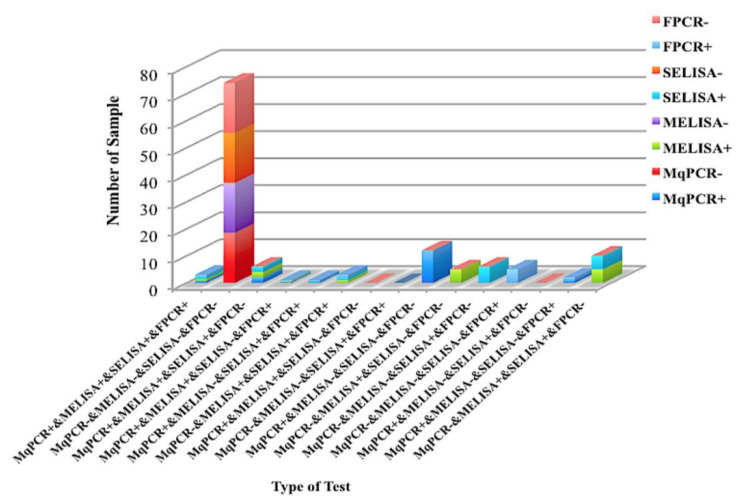
Distribution of 128 MIF milk samples categorized into fifteen quaternary groups based on their positive/negative status in MqPCR, MELISA, SELISA, and FPCR tests.

**Figure 12:**
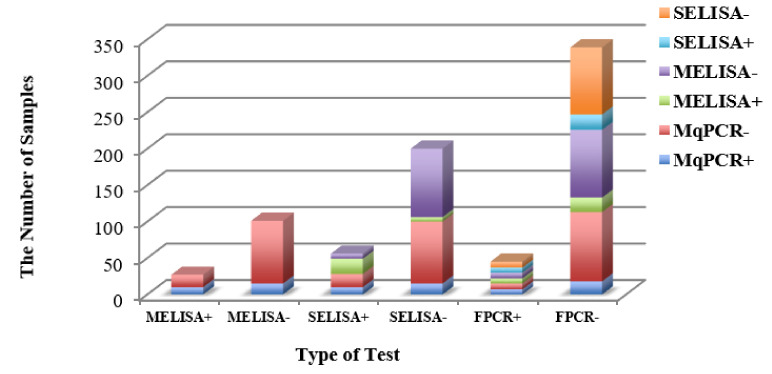
Distribution of 128 MIF milk samples classified into 24 binary groups based on the positive/negative status in MqPCR, MELISA, SELISA, and FPCR. The following categories had the greatest proportions: 74.22% Neg (MqPCR and FPCR), 73.44% Neg (MELISA and SELISA), 72.66% Neg (MELISA and FPCR), 71.88% Neg (SELISA and FPCR), 67.19% Neg (MqPCR and MELISA), and 66.41% Neg (MqPCR and SELISA).

According to the positive/negative status of samples from the four tests of MELISA, MqPCR, SELISA, and FPCR, the animals were stratified into 15 quaternary groups. A dominant number of samples (58%) were detected as being negative by all tests (MqPCR, MELISA, SELISA, and FPCR), compared to only 2% that were positive by all tests (Figure 11, Table 6). In another classification, the studied animals were classified into 24 binary groups based on the MAP status (positivity/negativity) of samples analyzed by each binary test of MqPCR and MELISA, MELISA and SELISA, MqPCR and FPCR, MELISA and FPCR, and SELISA and FPCR. Accordingly, Chi-square analysis was performed on each binary group. The proportion of samples in each category was as follows: 74.22% Neg (MqPCR and FPCR), 73.44% Neg (MELISA and SELISA), 72.66% Neg (MELISA and FPCR), 71.88% Neg (SELISA and FPCR), 67.19% Neg (MqPCR and MELISA), 66.41% Neg (MqPCR and SELISA), 7.8% Pos (MqPCR and MELISA), 13.3% Neg (MqPCR) and Pos (MELISA), 11.72% Pos (MqPCR) and Neg (MELISA), 16.4% Pos (MELISA and SELISA), 5.5% Neg (MELISA) and Pos (SELISA), 4.7% Pos (MELISA) and Neg (SELISA), 5.5% Pos (MqPCR and FPCR), 6.3% Neg (MqPCR) and Pos (FPCR), 14.06% Pos (MqPCR) and Neg (FPCR), 5.5% Pos (MELISA and FPCR), 6.3% Neg (MELISA) and Pos (FPCR), 15.6% Pos (MELISA) and Neg (FPCR), 5.5% Pos (SELISA and FPCR), 6.3% Neg (SELISA) and Pos (FPCR), and 16.41% Pos (SELISA) and Neg (FPCR). MELISA and SELISA were recognized as the most dependent tests (Chi-square: X^2^ (1, *n* = 128) = 62.57, *p* < 0.0001). However, the dependency gradually decreased between the following test groups: MqPCR and FPCR (*p* = 0.0048), MqPCR and MELISA (*p* = 0.0098), MELISA and FPCR (*p* = 0.0098), SELISA and FPCR (*p* = 0.0134), and MqPCR and SELISA (*p* = 0.0145) (Figure 12, Table 7).

## 5. Discussion

Our findings suggest that milk is a potent sample for screening paratuberculosis in sheep flocks. However, the sensitivity and specificity of milk tests, i.e., MqPCR and MELISA, might be affected by some factors, such as the type of milk samples (BTM or individual samples), the disease status of animals participating in the survey, and the selected gold standard for statistical analysis [28,29]. Our BTM analyses (MqPCR and MELISA) demonstrated that MAP positivity follows a seasonal pattern, which was proven through a longitudinal study on 14 sheep herds.

Our MIF-relevant studies showed that MELISA and SELISA had the highest levels of concordance among the tests (89.8%). Such agreements were previously recorded by two other studies about the evaluation of the kinetics of antibodies directed against MAP during the lactation period (R^2^ = 0.5358) [30] and the assessment of the efficiency of a multiplex bead-based immunoassay in the detection of MAP-immunogenic antigens in animals with JD (R^2^ = 0.572 to 0.756) [31].

We compared the efficiency of different gold-standard models to reach the highest sensitivity and specificity in detection of the true incidence rate of MAP in milk samples. We diagnosed that SELISA together with FPCR is one of the best practical reference models in individual-level milk assessments. However, each of them as an independent gold standard favors a specific milk assay (MqPCR or MELISA). The results suggest remarkable sensitivity and specificity of MELISA when the binary reference models were based on SELISA in isolation (SP: 0.94, SN: 0.75; *p* < 0.0001) and in combination with FPCR (SP: 0.95, SN: 0.61; *p* < 0.0001). In comparison, MqPCR had its highest specificity (SP: 0.87; SN: 0.4; *p* = 0.047) and sensitivity (SP: 0.84, SN: 0.47; *p* = 0.044) when the gold standards were based on SELISA + FPCR and FPCR, respectively. However, when FPCR was the standard model, the specificity of MELISA dropped slightly (SP: 0.72, SN: 0.67; *p* = 0.129). Our result is in accordance with another study that was conducted on BTMs from 21 dairy sheep flocks, evaluating the SP and SN of a modified milk ELISA in the detection of antibodies directed against MAP. A recent study that applied each of fecal PCR and serum ELISA as an independent reference test depicted that MELISA introduced a notable SP and SN when the reference test was based on SELISA (SP: 100%, SN: 72.7%) compared to when FPCR was the gold standard (SP: 46.7%, SN: 83.3%) [29]. Interestingly, in another comparative work on the evaluation of the efficiency of a high-yield fecal qPCR (YHDEqPCR) assay in the detection of MAP, the poorest level of agreement was seen between YHDEqPCR and milk ELISA (1%; *p* = 0.739), although the correlation between YHDEqPCR and milk qPCR was also insignificant (36%; *p* = 0.591) [14].

At the herd level (BTM), we developed a binary gold standard from both MqPCR and MELISA results that remarkably enhanced the sensitivity and specificity of each milk assay, i.e., MqPCR or MELISA. In order to assess whether or not this gold standard favors a specific milk assay, each of MqPCR and MELISA was used as an independent gold standard to evaluate the efficiency of the other diagnostic assay (MELISA or MqPCR). MqPCR offered a significantly greater sensitivity and specificity (SP: 1, SN: 0.95; *p* < 0.0001) than MELISA (SP: 0.89, SN: 0.59; *p* = 0.00035) when both MqPCR and MELISA were the reference models. We reached a similar SP but higher SN in MqPCR analysis than another BTM-MAP-detection study conducted on 21 sheep flocks under the condition that any of serum ELISA, milk ELISA, fecal PCR, and fecal culture was assigned as the gold standard (SP: 100%; SN: 25%) [29].

We found that MqPCR as a reference test induced a remarkable specificity (SP: 0.95, SN: 0.34; *p* = 0.037) to MELISA compared to when MELISA was the reference test and MqPCR was the diagnostic assay (SP: 0.79, SN: 0.5; *p* = 0.098).

Our results revealed that the incidence of MAP and antibodies against it were lower in the MIF level than the herd groups (BTM) [29,32,33,34]. MqPCR and MELISA results were concordant with each other by 75% and 50% in MIF and BTM levels, respectively. Accordingly, the positivity rates of MqPCR and MELISA were estimated to be 19.53–21.09% in the MIF group and 72.6–30.14% in the BTM group. Thus, 47% of BTM and 11.7% of MIFM cases were detected as being positive by MqPCR without showing a sufficient antibody titer for positivity by MELISA. The significant disagreement ratio (47%) between MqPCR and MELISA in the BTM group was probably due to the high proportion of intermittent MAP shedders in each herd, lack of environmental hygiene practices that inhibit the dissemination of MAP through the environment, the inequity of the animals that participated in the survey in terms of immune status, stages of disease, age, and genetic predisposition [16,28,35,36]. Furthermore, MqPCR IS900 is one of the most sensitive MAP-detection assays and can detect the lowest concentration of MAP in the MAP-shedder animals in the initial stages of JD. As an American survey on the assessment of the sensitivity of various MAP-detection approaches (fecal–milk culture and qPCR) demonstrated that qPCR IS900 can distinguish MAP-shedder cows via milk, colostrum, and feces more sensitively than the culture-based assays [9].

The results of BTM analyses (MqPCR and MELISA) on 73 samples from 59 herds showed an association between MAP-positivity rhythm and seasonal changes. We found that MqPCR and MELISA positivity have different seasonal patterns, in which the dominant number of positive MqPCR cases (45.21%) occurred in the spring. However, the highest number of MELISA-positive subjects (17.81%) was seen in the summer. This suggests that the number of MAP shedders increases in the spring due to seasonal breeding, possible sexual transmission of MAP, and the presence of animals shedding MAP via feces, which all corresponded to higher percentages of MqPCR-positive cases in the spring. However, humoral immunity due to MAP positivity was notable in the summer (MELISA). As a study on the detection of MAP in reproductive tissue showed that horizontal sex transmission increases the risk of MAP dissemination in infected rams, even though the lesions related to Johne’s disease are not developed in the reproductive tissues, MAP can be disseminated to the reproductive tissues [37,38,39,40,41,42]. Also, an Italian study on MAP seroprevalence in dairy sheep flocks evidenced that the rate of MAP seropositivity was enhanced in the spring and autumn (*p* < 0.071) [43].

However, our longitudinal study on 14 herds (BTMs) showed that the intensity of MAP-positivity, corresponding to lower TC values in MqPCR (ranged from 15 to 34 cycles), was more noticeable in the summer. A similar Canadian study on detecting MAP in milk and colostrum detected a higher proportion of MAP shedders in the summer by milk and colostrum qPCR than fecal and milk cultures [9]. Our longitudinal study also determined an increase in the number of positive MELISA cases in the summer. Even though some cases were MELISA-negative both in the spring and summer, the antibody titers against MAP had an upward trend in the summer and changes were remarkable in some cases (S/P% increased by between 1.3% and 60.4%). This finding is in accordance with the results of another study about the association of season of sampling with change of MAP antibody titers in BTM. This study also confirmed that the MAP antibody titer follows a seasonal pattern in milk samples: peaking in the summer and drastically dropping in the winter [23].

Our findings are limited by some factors, such as sample size, lack of communication with the herd’s owners regarding the health status of animals that participated in this survey and hygiene practices, and limitations in following up all herds in each season.

Our experiments on the efficiency of different reference standard models suggest that the type of gold-standard test remarkably affects the sensitivity and specificity of both MqPCR and MELISA tests. In MIF study, SELISA in isolation and together with FPCR introduced the best sensitivity and specificity to MqPCR and MELISA tests. Furthermore, MELISA and SELISA results were concordant in 75% of cases (*p* < 0.0001), and this proved that MELISA could be used as a predicting test in the MAP screening programs at the MIF level. Of course, this conclusion directly depends on some factors, such as the age, immune status, stage of disease and so forth of the animals that participated in the survey.

Our herd-level studies demonstrated that the results of MqPCR and MELISA together could form a practical binary reference model (gold standard) under the condition that there is no information on the health status of the herd’s animals. The results of both milk assays (MqPCR and MELISA) could represent how much potential each herd has to disseminate MAP through the environment and transmit the disease to other herds. The BTM-level studies depicted that MqPCR is more sensitive and specific (*p* < 0.0001) than MELISA (*p* = 0.00035). However, the agreement between the results of both milk assays was statistically significant when the *p*-value was adjusted to 0.1 (*p* = 0.083); this was equal to 50% concordance. This suggests that a high proportion of animals in each herd were MAP shedders.

Our studies on 73 sheep herds indicated that MAP positivity follows a seasonal pattern. The number of positive MqPCR cases (45.21%) peaked in the spring, while the trend of MELISA positivity was notable (17.81%) in the summer. However, the result of the longitudinal study on 14 herds showed that the lower TC values, corresponding to stronger positivity, are most obvious in the summer. This may suggest that a large number of MqPCR-positive cases in the spring could be MAP shedders.

The results obtained in this study highlight the risk of the transmission of this pathogenic mycobacterium to the community. A higher exposition to MAP, which causes a persistent infection in its host [44], may lead to trigger not only inflammatory bowel diseases including Crohn’s disease [44,45], but also different autoimmune diseases, as it has been previously associated with in several studies [46,47,48,49,50,51,52,53,54,55]. This should motivate reflection by legislators and encourage the adoption of the best policies to lower the leakage of this bacterium into communities.

## Figures and Tables

**Table 1:** The number of milk samples in each category of BTM and MIF and date of sampling.

Number of Samples	Type of Sheep Milk Sample	Date of Sampling
73	Bulk tank milk (BTM)	23.07.2018 to 25.07.2019
128	MAP-infected flock milk (MIFM)	10.04.2019 to 23.09.2019

**Table 2:** Distribution of BTMs from 14 sheep herds (28 BTMs) in four categories based on MqPCR status (positive/negative) in a longitudinal study.

Samples	TC%
MqPCR was first negative, then positive	21.43%
MqPCR was positive in both rounds of sampling	28.57%
MqPCR was negative in both rounds of sampling	7.14%
MqPCR was first positive, then negative	42.86%

**Table 3:** Comparison between MELISA sample-to-positive ratio (S/P%) of 16 BTMs before and after fractionating milk samples.

Sample No.	Whole BTM S/P%	Whey BTM S/P%
6	19.33609959	35.64315353
9 *	−0.497925311	−0.082987552
11	20.08298755	22.44813278
12	11.90871369	27.63485477
13	29.70954357	43.15352697
14	14.97925311	22.98755187
28	11.24481328	15.97510373
30	2.033195021	2.780082988
33	21.82572614	30.45643154
37	9.211618257	9.543568465
38	3.278008299	4.647302905
41	10.66390041	14.10788382
53	11.0373444	15.72614108
73	47.71784232	60.24896266
80	25.6846473	39.04564315
82	21.45228216	27.38589212

* Sample 9 was a known negative commercial BTM.

**Table 4:** MELISA status (positive/negative) of BTMs from 14 sheep herds in a longitudinal study.

Samples	Results (%)
MELISA was first negative, then positive	28.57%
MELISA was positive in both rounds of sampling	14.28%
MELISA was negative in both rounds of sampling	57.14%

**Table 5:** Distribution of 73 BTMs based on the positivity/negativity status in MqPCR and MELISA tests and corresponding *p*-values.

	MELISA+	MELISA−
MqPCR+	19	34
MqPCR−	3	17
*p*-value (p < 0.1) *	0.083	
*p*-value (p < 0.05)	Insignificant (IS)	

* Since the dependency was insignificant at *p* < 0.05, the statistical significance was adjusted for *p* < 0.1.

**Table 6:** Distribution of 128 MIF milk samples in fifteen quaternary groups based on positive/negative status in MqPCR, MELISA, SELISA, and FPCR tests.

Type of Tests	Number of Samples
MqPCR+ and MELISA+ and SELISA+ and FPCR+	3
MqPCR– and MELISA– and SELISA– and FPCR−	74
MqPCR+ and MELISA+ and SELISA+ and FPCR−	6
MqPCR+ and MELISA+ and SELISA− and FPCR+	1
MqPCR+ and MELISA− and SELISA+ and FPCR+	1
MqPCR− and MELISA+ and SELISA+ and FPCR+	3
MqPCR+ and MELISA+ and SELISA− and FPCR−	0
MqPCR− and MELISA− and SELISA+ and FPCR+	0
MqPCR+ and MELISA− and SELISA− and FPCR−	12
MqPCR− and MELISA+ and SELISA− and FPCR−	5
MqPCR− and MELISA− and SELISA+ and FPCR−	6
MqPCR− and MELISA− and SELISA− and FPCR+	5
MqPCR+ and MELISA− and SELISA+ and FPCR−	0
MqPCR+ and MELISA− and SELISA− and FPCR+	2
MqPCR− and MELISA+ and SELISA+ and FPCR−	10
Total number of samples	128

**Table 7:** Chi-square analysis and the distribution of 128 MIF milk samples classified into 24 binary groups based on the positive/negative status in MqPCR, MELISA, SELISA, and FPCR and corresponding *p*-values.

	MELISA+	MELISA-	SELISA+	SELISA−	FPCR+	FPCR−
MqPCR+	10	15	10	15	7	18
MqPCR−	17	86	18	85	8	95
*p*-value (*p* < 0.05) *	0.0098		0.0145		0.0048	
MELISA+			21	6	7	20
MELISA−			7	94	8	93
*p*-value (*p* < 0.05) *			*p* < 0.00001		0.0098	
SELISA+					7	21
SELISA−					8	92
*p*-value (*p* < 0.05) *					0.0134	

* Statistical significance was adjusted for a *p*-value of < 0.05.

## Abbreviations

JD	Johne’s disease
MAP	*Mycobacterium avium subsp. Paratuberculosis*
BTMs	Bulk tank milk samples
qPCR	Quantitative polymerase chain reaction
IS900	Insertion sequence 900
ELISA	Enzyme-linked immunosorbent assay
MqPCR	Milk qPCR
MELISA	Milk ELISA
SELISA	Serum ELISA
FPCR	Fecal PCR
MIF	MAP-infected flock
MIFMs	MAP-infected flock milk samples
PBS	Phosphate-buffered saline
HPC	Hexadecylpyridinium chloride
S/P%	Sample-to-positive ratio
ROC	Receiver operating characteristic
AUC	Area under the curve
TC	Threshold cycle
Sensitivity	SN
Specificity	SP
Reference	Reference
Pos	Positive
Neg	Negative
